# Both family and peer relationships matter in late adolescence: a network analysis of loneliness and social connection in a UK cohort

**DOI:** 10.1136/bmjph-2025-004053

**Published:** 2026-05-21

**Authors:** Kathryn E Bates, Lara Acosta Siljeström, Amanah Bokhari, Leandra Gyekye, Jennifer Y F Lau, Lauren Turner, Delia Fuhrmann

**Affiliations:** 1 Institute of Psychiatry, Psychology and Neuroscience, King’s College London, London, UK; 2 Queen Mary University of London, London, UK

**Keywords:** Adolescent, Public Health, Sociodemographic Factors

## Abstract

**Introduction:**

Adolescents from low socioeconomic backgrounds report higher rates of loneliness than their peers from high socioeconomic backgrounds. Reasons for this vulnerability remain unclear. We investigated how peer bullying, family conflict, loneliness and social connection relate at different levels of socioeconomic marginalisation.

**Methods:**

Using data from the UK Household Longitudinal Survey (N=2531), we captured loneliness and social connection at 16–24 years and bullying and family conflict at 10–15 years. Network analysis tested item-level relationships while controlling for other variables. Previous investigations have not accounted for the exclusionary characteristics of low socioeconomic status, that is, socioeconomic marginalisation. We calculated a socioeconomic marginalisation score using publicly available data to assess an individual’s income relative to their local community. We then compared loneliness and social connection networks to investigate potential differences.

**Results:**

The most central items in the full sample network were talking to family about worries, talking to friends about worries and relying on friends (16–24 years), as well as peer bullying (10–15 years). Distinct facets of loneliness were linked to specific social connection and bullying experiences. For example, feeling left out was associated with feeling let down by friends (w=−0.10) and feeling less understood by friends (w=−0.05). Whereas feeling isolated was associated with feeling less understood by family (w=−0.11) and less understood by friends (w=−0.04). Peer bullying related to feeling more left out (w=0.05) and more isolated (w=0.04). Some associations (eg, feeling less let down and less criticised by family) were stronger in the high socioeconomic marginalisation group, who also reported more severe bullying.

**Conclusion:**

Evidence suggested family relationships remain important in late adolescence. Findings highlight the value of item-level dynamics for targeted support. In the context of public health policy, prevention and intervention measures should be tested that prioritise earlier bullying experiences and reflect the importance of both family and peer connection in late adolescence.

**WHAT IS ALREADY KNOWN ABOUT THE TOPIC**

- Adolescents from low socioeconomic backgrounds often report higher levels of loneliness.
- Previous literature testing the relationship between socioeconomic status and loneliness is often limited to (1) single item or composite scores of loneliness and (2) socioeconomic status measures that do not account for marginalisation.

**WHAT THIS STUDY ADDS**

- This study shows that specific facets of loneliness are linked to distinct aspects of social connection and past peer bullying.
- Family and friend relationships, as well as past peer bullying, remain important in loneliness and social connection networks even in late adolescence.
- Adolescents experiencing high socioeconomic marginalisation are more likely to report more severe bullying, and some relationships between specific aspects of loneliness and social connection were stronger in this group.

**HOW THIS STUDY MIGHT AFFECT RESEARCH, PRACTICE OR POLICY**

- The study shows the value of investigating item-level dynamics in loneliness, social connection and bullying to identify suitable targets for support.
- Confirmatory and intervention studies should consider the multidimensionality of loneliness and social connection.
- Policies addressing adolescent psychosocial health should integrate family and peer relationships, particularly in socioeconomically marginalised groups, to address inequalities.

## Introduction

 The experience of loneliness, when our need for social relationships or emotional companionship within relationships is not met^1^, is universal. As social beings, the experience of transient loneliness encourages us to engage with others.^2^ However, when loneliness becomes prolonged and feels inescapable, it increases the risk of cardiovascular disease, depression, anxiety and early mortality.^3^,^5^ Both the UK government and WHO have highlighted loneliness as a pressing public health issue.^6 7^

Adolescents (aged 10–24 years) may be particularly likely to experience prolonged loneliness. Large international, cross-sectional studies have suggested loneliness peaks in late adolescence, particularly around the age of 19 years.^8^,^11^ To understand why adolescents later in development might be at greater risk of loneliness, we need to consider potential developmental origins. Adolescents may be at heightened risk of exclusion and interpersonal conflict compared with other age groups. During late adolescence, social networks expand^12^ alongside major life transitions in education, work and independence.^13^ The nature of relationships changes; intimate relationships are explored by some,^14^ and many have greater responsibility at home and with family members.^15 16^ The heightened sensitivity to social cues among emotional and social instability can make exclusion more acute. These internal and external transitions are linked to increased loneliness.^17^

Some adolescents are at greater risk of severe (prolonged/overwhelming) loneliness than others. Studies have shown that adolescents from lower socioeconomic status (SES) backgrounds report higher rates of loneliness compared with those from higher SES backgrounds.^18^,^21^ SES refers to an individual’s economic and social status, typically measured using one or a combination of income, education and occupation. Many people are in a vulnerable position: over one in five people is currently living in poverty in the UK alone.^22^ Cross-national studies in the UK, Norway and Kenya show links between poorer family finances, unemployment and greater loneliness.^1823^,^25^ The evidence in this area is not fully conclusive: a recent systematic review pointed out that some studies did not find significant associations between loneliness and socioeconomic factors.^26^ SES is a complex concept that can be difficult to operationalise; research investigating this relationship needs robust indicators.

The mechanisms as to *why* young people from low SES backgrounds may be at higher risk of loneliness are currently unclear. Qualitative research shows adolescents (aged 18–24 years) from low SES backgrounds often feel excluded, misunderstood and pressured to be accepted. Specifically, they reported that worrying about money and lack of money led them to feel less connected to others.^27^ The current SES measures used to investigate the relationship between SES and loneliness do not account for the social aspects of marginalisation. For example, measuring only household income does not reveal whether the individual’s income is low relative to those in the local community, which would reflect socioeconomic marginalisation. Recent theoretical work has framed loneliness as a social justice issue, stipulating that the marginalisation of low SES groups, i.e., the relegation of individuals to the periphery of society, may intrinsically put them at greater risk of loneliness.^28^ Research accounting for socioeconomic marginalisation is needed to understand *why* some adolescents are at greater risk.

The exclusionary characteristics of socioeconomic marginalisation can include bullying experiences. Bullying, defined as repeated abuse where the individual cannot defend themselves^29^, is common in adolescence: around 30% of those aged 12–17 years (n=317 869) surveyed said they had experienced at least one instance of bullying in the last month.^30^ Bullying is experienced more often in young people from low SES backgrounds than those from high SES backgrounds.^31^ Longitudinal evidence has shown that peer bullying and social isolation experienced more often in childhood (age 12 years) predicted higher loneliness at the age of 18 years.^23^ This link between earlier bullying and later loneliness is thought to be because individuals are statistically more likely to feel rejected, a lack of belonging and struggle to trust others, which, in turn, makes it more likely that they withdraw and find it hard to maintain relationships.^32^,^34^ This social sensitivity is not limited to peers; family dynamics can also play a role. Supportive families can buffer against loneliness.^35^ Research examining both family and peer relationship dynamics in the context of different levels of SES is needed to disentangle these relationships.

On the other hand, social connection is thought to protect against loneliness.^36^ Social connection is typically defined across structural, functional and quality dimensions,^37^ whereas loneliness reflects the subjective, personal experience of dissatisfaction with these relationships. Evidence highlights the importance of supportive peer and family relationships, but the relationship between social connection and loneliness in adolescents experiencing socioeconomic marginalisation remains unclear.^38^

In this study, we investigated how peer bullying and family conflict in early adolescence (age 10–15 years) is related to loneliness and social connection in late adolescence (age 16–24 years) in low and high socioeconomic marginalisation groups to address these gaps in the literature. SES is a multifaceted construct that encompasses finances, education, occupation and community resources.^39^ Here, we focus on income to assess an individual’s socioeconomic marginalisation relative to those in their local community. We used publicly available data of average income for 390 local government areas in the UK. Importantly, this method provides a community-level index that emulates the country-level Gini coefficient of income inequality,^40^ but at a more fine-grained local area level. This index can be readily applied in future research across other datasets, regions and countries to enhance the generalisability of findings. This research aligns with a socioecological systems approach recognising multiple interacting developmental systems^41 42^ and tests social justice theories of loneliness.^28^ We adopted an exploratory, theory-building approach using network analysis. We aimed to examine links between peer bullying and family conflict in early adolescence and later loneliness and social connection, and whether these differed by high and low socioeconomic marginalisation.

Network analysis is exploratory by design. Therefore, our hypotheses were non-directional. We expected distinct facets of loneliness to relate differently to friend and family social connection, with bullying and family conflict significantly associated with distinct facets of loneliness, and ‘feeling left out’ expected to be central to the network for adolescents experiencing high socioeconomic marginalisation. This addresses gaps in the research identified by policymakers^6^ and should inform confirmatory research and future interventions for adolescents in different economic contexts.

## Methods

### Design

We employed network analysis to investigate item-level dynamics between past experiences of bullying and family conflict (age 10–15 years) and loneliness and social connection later in adolescence (age 16–24 years). Network approaches propose that mental health conditions and related constructs arise from interacting systems or experiences, rather than a single underlying cause, enabling data-driven investigation of complex psychological dynamics.^43 44^ Emerging work has used network analysis to investigate constructs beyond psychopathology, including how adversities such as bullying and family dysfunction inter-relate^45^ and how loneliness relates to different facets of well-being, such as anxiety.^25 46^ Network analysis allows examination of unique item-level associations while controlling for others, highlighting specific prevention and intervention targets. We first fit a network to the full sample. We then calculated an intuitive socioeconomic marginalisation score by combining household income with local authority averages (detailed in the Methods section) and split participants into high and low socioeconomic marginalisation groups for comparison.

### Patient and public involvement

We recruited two youth co-researchers onto the project aged 16–18 years. They have contributed to each stage of the research. They have consulted on the design, conceptualisation of key concepts, development of research questions and they have provided lived experience reflections on the research findings here. The youth co-researchers reflected on whether the findings were surprising to them, whether the findings resonated with their experiences as a young person and whether any of the language was stigmatising (detailed in box 1). The youth co-researchers were paid for their time and are coauthors of this paper.

**Youth co-researchers' reflections**

We asked our youth co-researchers to reflect on whether they found any of the findings surprising or different to what they might expect; whether the findings resonated with their experiences as a young person and whether any of the language was stigmatising. They reported that they were surprised that adolescents in the high socioeconomic marginalisation group did not report higher levels of loneliness compared to the low socioeconomic marginalisation group. They also said that the findings led them to reflected that loneliness is not only related to personal social experiences but is also related to broader structural factors. They reported that the following findings resonated with them:

- Earlier experiences of bullying can shape later experiences of social connection and loneliness.
- The importance of feeling able to talk about worries with friends and family, especially during exam periods of life transitions when young people can feel left out and alone.

They reported that the term "victimisation" can be stigmatising, and we have removed references to this in the article. They suggested that a glossary of statistical terms would make the article more accessible to a wider audience; we have included this in online supplemental table S1.

### Sample

We used data from the UK Household Longitudinal Survey (UKHLS): a large-scale panel survey on family life, income, education, employment and well-being with participants aged 10 years old to over 100 years old (N~100 000).^47^ The University of Essex Ethics Committee has approved all data collection for the Understanding Society main study and innovation panel waves, including asking for consent for all data linkages except health records. Data from wave 11 (collected between January 2019 and May 2021) was downloaded in February 2024, and variables were retrieved from the youth, individual responses, household responses and stable characteristics files. We also acquired Special Licence Access to local authority district code data with approval granted in February 2024. Details on accessing UKHLS data can be found on the UK Data Service website: https://ukdataservice.ac.uk.

Participants aged 16–24 years were selected from the main panel survey, and their corresponding data from the youth survey were selected from ages 10–15 years. This resulted in a sample of 12 621 participants with data for at least one relevant variable. Network analysis uses listwise deletion to handle missing data; therefore, the final sample included was 2531 participants with data for all variables. With respect to follow-up rates, we checked this for participants who were old enough to have participated from Wave 1 to 11 (n=1183). Of these, 31% were lost, which is a low attrition rate over 10 years. Missing data patterns are detailed in online supplemental figure S1. Additionally, participants were excluded if z-scores on items were +/−5, to identify potential errors in data handling rather than natural variation in the data (n=12 on physical bullying item, no other exclusions). We retrieved sex and ethnicity information from the stable characteristics file, which contains the latest available information across all waves for these variables. Self-reported sex assigned at birth is labelled male or female if the data across waves consistently suggests the same category. In this sample, 56% were classified as female and 44% as male. UKHLS recorded participants’ sex as ‘inconsistent’ if the information was not consistent between waves and their forenames did not indicate male or female (n=2).

Participants’ mean gross household income was £66 539 (SD = £62 357). The Office for National Statistics (ONS) reports the average gross household income per head. In 2021, the average gross household income per head reported by the ONS was between £17 636 and £22 213 across the devolved nations in the UK.^48^ For comparability, UKHLS participants’ mean gross household income divided by number of people living in the participants’ household was £17 499 (SD = £15 284). Sex, ethnicity, age and proportion of participants living with parents for the full sample and by group are presented in table 1. As detailed in table 1, this sample is representative of the UK population with respect to gender and ethnicity.^49^ Note that the high and low socioeconomic marginalisation sample sizes do not add up to the full sample due to missing data in the grouping variable (N missing=129).

**Table 1:** Sample size and percentage of sample per level in sex, ethnicity, age and living with parents in the full sample and by group

Demographics	Full sample (n=2531) N (%)	High SEM group (n=1657) N (%)	Low SEM group (n=745) N (%)
Sex			
Female	1119 (56)	915 (38)	414 (17)
Male	1422 (44)	740 (31)	331 (14)
Ethnicity			
White	1932 (76)	1182 (49)	648 (27)
Asian	381 (15)	300 (13)	51 (2)
Black	101 (4)	83 (3)	15 (1)
Mixed	110 (4	75 (3)	29 (1)
Other	19 (1)	17 (1)	–
Missing	14 (.50)	NA	NA
Age in years			
16	299 (12)	237 (10)	56 (2)
17	310 (12)	216 (9)	76 (3)
18	381 (15)	259 (11)	99 (4)
19	290 (11)	203 (9)	73 (3)
20	306 (12)	203 (8)	81 (3)
21	289 (11)	163 (7)	101 (4)
22	264 (10)	163 (7)	86 (4)
23	244 (10)	133 (6)	100 (4)
24	160 (6)	79 (3)	74 (3)
Living with parents			
Yes	2286 (90)	1500 (62)	651 (27)
No	257 (10)	157 (7)	94 (4)

Sex and ethnicity data were retrieved from the stable characteristics file generated by UKHLS. ‘White’ includes responses to: British/ English/Scottish/Welsh/Northern Irish, Irish, Gypsy or Irish traveller and any other white background. ‘Asian’ includes responses to: Indian, Pakistani, Bangladeshi, Chinese and any other Asian background. ‘Black’ includes responses to: Caribbean, African and any other black background. ‘Other’ includes responses to: Arab and any other ethnic group. ‘Missing’ refers to missing responses. Values ≤10 are not reported to prevent statistical disclosure.

SEM, socioeconomic marginalisation.

### Measures

Descriptive statistics for all measures are presented in the online supplemental figure S2. Variables were entered into the network at the item-level, rather than constructing composite scores. We have indicated which variables were entered into the network under each subsection describing the measures.

#### Loneliness

Loneliness was measured using the UCLA (University of California, Los Angeles) 3-item Loneliness Scale^50^ with the items ‘How often do you feel you lack companionship?’, ‘How often do you feel isolated from others?’ and ‘How often do you feel left out?’. There were three response options: 1= ‘hardly ever or never’, 2= ‘some of the time’ and 3= ‘often’. Cronbach’s alpha for these items ranged from 0.70 to 0.82. Each of the three loneliness items was entered into the network.

#### Social connection

We retrieved data from the ‘Self-Completion Adult Social Support’ module from the UKHLS dataset to capture the three social connection factors outlined by Holt-Lunstad^36^: structural (relationships/roles), functional (perceived support) and quality (positive/negative aspects). Function and quality were assessed with items on perceived availability of support and affective relationship aspects (table 2). Structure was implicit; only those reporting having immediate family and friends completed the questionnaire. Analysis included family and friends items. Only 5% of the sample responded to the spouse questionnaire; therefore, this was not included. Responses ranged from 1= ‘a lot’ to 4= ‘not at all.’ Positively phrased items were reverse coded so that for all items, higher scores indicate greater social connection. Cronbach’s alpha ranged 0.77–0.79. Each of the six items for social connection with family and each of the six items for social connection with friends was entered into the network.

**Table 2:** Social connection items asked separately about family and friends with the corresponding level of social connection (as conceptualised by Hold-Lunstad)^36^

Item	Level of social connection
‘How much do they understand about the way you feel about things?’	Quality
‘How much can you rely on them if you have a serious problem?’	Function
‘How much can you open up to them if you need to talk about your worries?’	Function
‘How much do they criticise you?’	Quality
‘How much do they let you down when you are counting on them?’	Function
‘How much do they get on your nerves?’	Quality

#### Peer bullying and family conflict aged 10–15 years

We retrieved variables from the youth survey (administered aged 10–15 years) on bullying and family conflict from the preceding waves of data collection (waves 1–10). The mean score from responses aged 10–15 years was computed for each variable.

We included the three bullying items available. Physically bullied (‘How often are you physically bullied at school?’) and bullied in non-physical ways (‘How often are you bullied in other ways at school?’) were rated from 1= ‘never’ to 4= ‘a lot (a few times every week)’. In the bullying item from the Strengths and Difficulties Questionnaire, participants rated the statement ‘other children or young people pick on or bully me’ from 1= ‘not true’ to 3= ‘certainly true’. Cronbach’s alpha ranged from 0.70 to 0.75.

Family conflict was captured by the following two items: ‘How often do you quarrel with (mother/father)?’. Cronbach’s alpha was good at 0.72. Each bullying (three) and family conflict (two) items were entered into the network.

#### Socioeconomic marginalisation

Socioeconomic marginalisation was captured by computing participants’ gross annual household income per head relative to the average gross income per head in their community. The UK is made up of 390 local authorities (see map in online supplemental figure S3), which are areas defined by local governments, for example, Coventry, London Borough of Barnet, Fife (Scotland), Mid Ulster (Northern Ireland). We used publicly available data on gross annual income per head. Average gross income and household size were available from different sources depending on location. Data for our main analysis were collected by UKHLS between 2019 and 2021; we used data from 2020 or the closest available year depending on availability. Gross income for all local authorities across England, Wales, Scotland and Northern Ireland was accessed from the ONS.^51^ When data was collected for UKHLS, Corby and East Northamptonshire had seven different local authority districts; they are now inactive and are combined into one. Gross income for the seven districts was available for 2018 and used here.^52^

Gross annual household income per head accounts for taxes and benefits and does not deduct specific household expenses like pensions or child support payments like net annual household income would. However, net household income was not publicly available for local authorities in Scotland and Northern Ireland. For comparability, we have therefore used gross annual income. We calculated a socioeconomic marginalisation score accounting for the number of people living in the household using the following formula: (gross annual household income/number in household)/average gross income per head in local authority.

Using this formula, a score below 1 reflects socioeconomic marginalisation relative to the local community. We can calculate a hypothetical example using data from the ONS. The average gross household annual income per head in Coventry is £15 097. A participant with a gross household annual income of £26 000 living in a household of two people would score 0.86, indicating socioeconomic marginalisation relative to their local community: (26 000/2)/15 097=0.86.

We then grouped participants based on this score: participants scoring less than one were grouped into the high socioeconomic marginalisation group, and participants scoring above 1 were grouped into the low group. For the group comparisons analysis, 129 participants were excluded due to missing income data, which resulted in 1657 (69%) in the high economic marginalisation group and 745 (31%) in the low economic marginalisation group. The socioeconomic marginalisation score calculated here can be reproduced using local community average household income, participant household income and the community they live in (e.g., county council, borough, country). The marginalisation score can be calculated for other datasets, regions and countries in the same manner, where average income metrics are available.

### Data analytic procedure

Network analysis allows us to identify relationships between specific variables (nodes) and the relationships between them (edges), while controlling for all other nodes in the network. To understand relationships at the item level rather than average scores, 20 nodes were entered into the network as detailed in Measures section. Weak edges are set to zero by setting a tuning parameter, meaning that spurious partial correlations are less likely to affect the network structure, and the remaining edges can be interpreted meaningfully. We set the tuning parameter to the default value (gamma=0), which still removes edges that do not improve fit but is less conservative than higher parameters. Our data were non-normally distributed and contained both ordinal and categorical data; we fitted a non-regularised Gaussian graphical model, which handles these properties well.^53^ Only participants with data on all the items can be included in the network analysis (complete pairwise cases). To test the network’s accuracy and stability, we applied non-parametric bootstrapping with 2000 samples (online supplemental figure S4). We have included a glossary of statistical terms in the online supplemental table S1.

Centrality indices allow us to estimate a node’s influence on the network: strength (overall connectedness), betweenness (importance in pathways), closeness (distance to others) and expected influence (connectivity accounting for positive and negative relationships).^54^ Expected influence captures both positive and negative relationships, and we focus on this index in the results, with other indices reported in online supplemental figure S5.

To explore how nodes clustered together, we used Exploratory Graphical Analysis with the Walktrap algorithm to estimate clusters in the network. We can then test whether there are specific nodes that bridge two or more clusters; identifying bridge nodes could guide targets for support. In this study, to avoid overinterpreting weaker nodes, we used the top 80th percentile of bridge nodes.^55^

To investigate differences between groups, we fit a network to the high and low socioeconomic marginalisation groups and then conducted a Network Comparison Test (a permutation test with 1000 iterations) to compare the overall strength of the network, the structure of the network and whether there were differences between edges.^56^ To aid interpretation of the network comparison between high and low socioeconomic marginalisation groups, we conducted χ^2^ tests adjusted for multiple comparisons on proportions of demographics between the two groups for sex (male, female), ethnicity (white, Asian, Black, other) and living with parents (yes or no). We also conducted *Welch’s* t-tests on item scores for all variables with Bonferroni corrections for multiple comparisons, 20 in this instance.

We conducted two sensitivity checks. First, we explored the effect of the COVID-19 pandemic as data were collected between January 2019 and May 2021. We split the sample into those who participated before the first UK COVID-19 lockdown (participating on or before 29 February 2020; n=1591) and those who participated after the lockdown and therefore during the pandemic (participating after 29 February 2020; n=940). We conducted another network comparison test to assess differences between the two groups’ networks. The second sensitivity check was to split the groups into low, middle and high levels of socioeconomic marginalisation. Because the network comparison test is designed for pairwise comparisons only, we conducted three separate network comparison tests (low vs middle, low vs high and mid vs high) to evaluate differences between the three groups. We applied Bonferroni corrections for multiple comparisons to p values, three in this instance.

Our main analysis script is available on the Open Science Framework: Analyses were conducted in RStudio V.4.3.1 using *bootnet*^57^ (network estimation), *qgraph*^58^ (visualisation), *EGAnet*^59^ (clusters) and *NCT*^56^ (network comparisons).

### Data availability

The UKHLS dataset is available through application with the UK Data Service: https://ukdataservice.ac.uk.

## Results

We used network analysis to examine relationships between peer bullying and family conflict (ages 10–15) and loneliness and social connection (ages 16–24). The sample was split into high and low socioeconomic marginalisation groups to compare network structure, strength and edges. Network accuracy, assessed via bootstrapping (online supplemental figure S4), showed greater variability in negative edges, though CIs were within expected ranges. Correlation stability (CS) was used to assess network stability; the optimal coefficient is 0.75 or above. Stability for strength and expected influence was optimal (*CS*s=0.75), stability for closeness (*CS*=0.672) and bridge strength (*CS*=0.595) was acceptable and stability was poor for betweenness (*CS*=0.205), bridge expected influence (*CS*=0.439), bridge closeness and bridge betweenness (*CSs=*0.05). Betweenness and bridge nodes should therefore be interpreted with caution.

### Dynamic relationships between peer bullying and family conflict aged 10–15 years and social connection and loneliness aged 16–24 years

**Figure 1:**
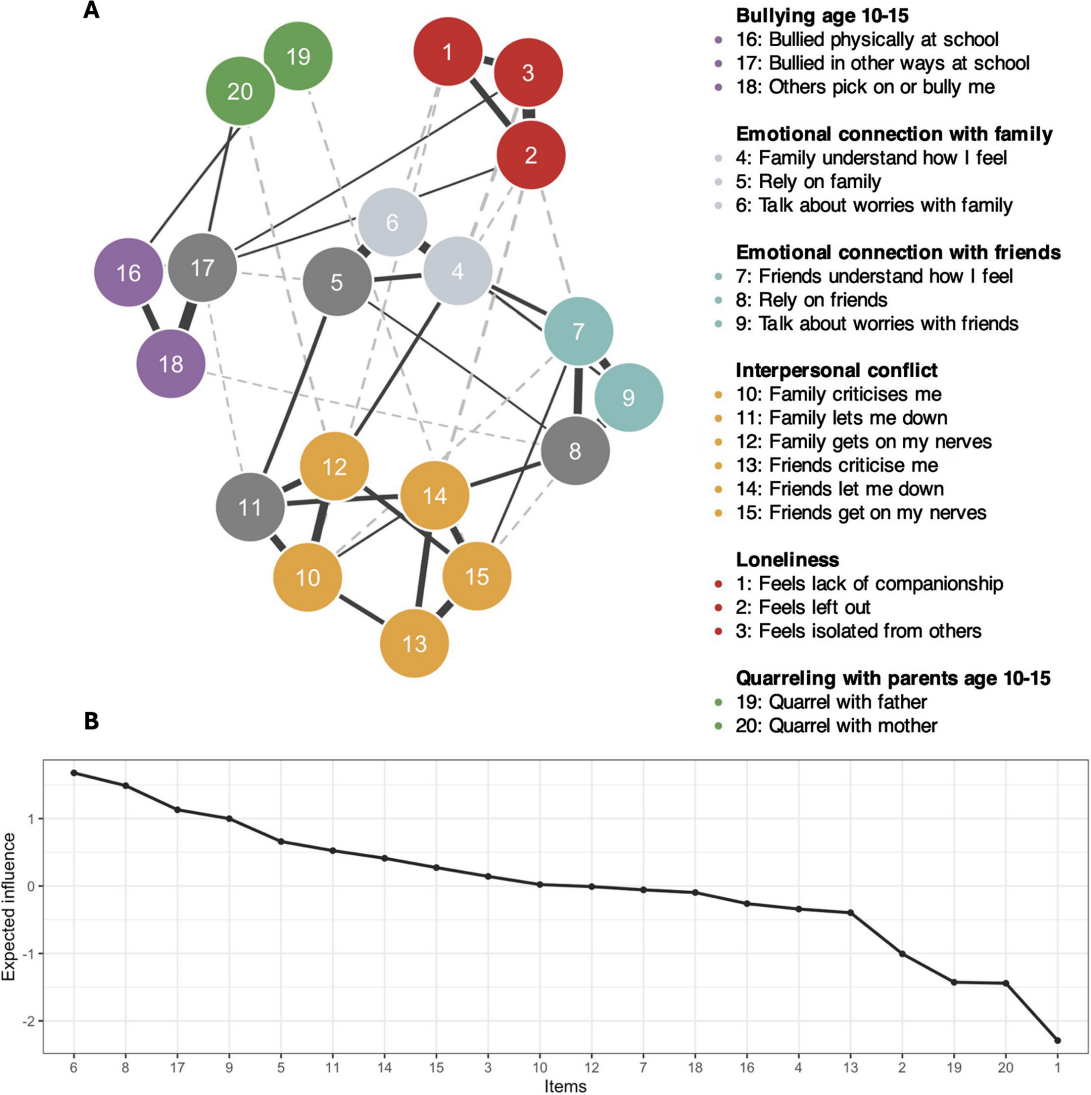
Network of past bullying and family conflict (aged 10–15 years) and current loneliness and social connection (aged 16–24 years) visualised in the top panel (**A**). Positive relationships are visualised as full, black lines; negative relationships are visualised as dashed, grey lines. The more saturated the line, the stronger the relationship. Bridge nodes are visualised in dark grey (items 5, 8, 11 and 17). Expected influence is visualised in the bottom panel (**B**); items are organised on the x-axis from highest to lowest expected influence.

Our first aim was to understand the relationships between bullying and family conflict aged 10–15 years and loneliness and social connection aged 16–24 years. The network and expected influence are visualised in figure 1 (full centrality statistics; online supplemental figure S5) and the edge weights matrix is visualised in online supplemental figure S6.

Clusters were conceptualised as follows: loneliness, emotional connection with family, emotional connection with friends, interpersonal conflict, bullying (aged 10–15 years) and quarrelling with parents (aged 10–15 years). According to the expected influence metric, the most central items in the network were talking to family about worries, relying on friends, talking to friends about worries aged 16–24 years and being bullied in non-physical ways at school from age 10 years to 15 years. Relying on family, relying on friends, feeling let down by family and being bullied in non-physical ways at school aged 10–15 years were identified as bridge nodes; however, this should be interpreted with caution due to low stability of this metric. These results highlight a distinction between social connection with family and friends, as well as the relevance of both current social connection and past bullying in the network.

Distinct associations emerged between loneliness items and facets of social connection and bullying, after controlling for all other edges in the network. We first consider each of the loneliness items. Lacking companionship was linked to less ability to talk about worries with family (w=−0.07) and more family criticism (w=0.06). Feeling left out was related to being let down by friends (w=−0.10), feeling less understood by friends (w=−0.05) and family getting on nerves (w=−0.05). Feeling isolated was associated with feeling less understood by family (w=−0.11) and feeling less understood by friends (w=−0.04). Being bullied more often by peers in non-physical ways at ages 10–15 years was associated with feeling left out (w=0.05) and isolated (w=0.04). These findings demonstrate the value of item-level analysis: past peer bullying and current social connections with both friends and family show distinct relationships with different facets of loneliness.

Feeling less understood by family was associated with feeling more able to talk about worries with friends (w=−0.05) and feeling more criticised by family was associated with feeling more understood by friends (w=−0.06). This suggests a possible trade-off between turning to family versus friends in late adolescence.

Quarrelling with parents (ages 10–15 years), here termed family conflict, was not significantly associated with loneliness items in the network. These items showed differential associations with emotional connection with friends and past bullying (see figure 1). For example, quarrelling with mothers more often was associated with the family getting on nerves more (w=−0.07), and quarrelling with fathers was associated with friends getting on nerves more (w=−0.06). Physical peer bullying was associated with quarrelling more with fathers (w=0.07), while non-physical peer bullying related to quarrelling more with mothers (w=0.08).

Overall, lacking companionship and feeling isolated were associated with family connection factors, while feeling left out was associated with both family and friend connection factors. Earlier peer bullying was associated with later loneliness for items feeling left out and feeling isolated. This indicates social connection with both friends and family remained important in late adolescence.

To test differences by socioeconomic marginalisation, we fit networks for high and low groups and conducted a Network Comparison Test. This compares network structure, global strength and edge strength: the structure reflects the pattern of connections, global strength the sum of absolute edge weights and edge strength the magnitude of the strength between pairs of nodes. As this was an exploratory analysis, we did not correct significance tests between edges for multiple comparisons.^56^

**Figure 2:**
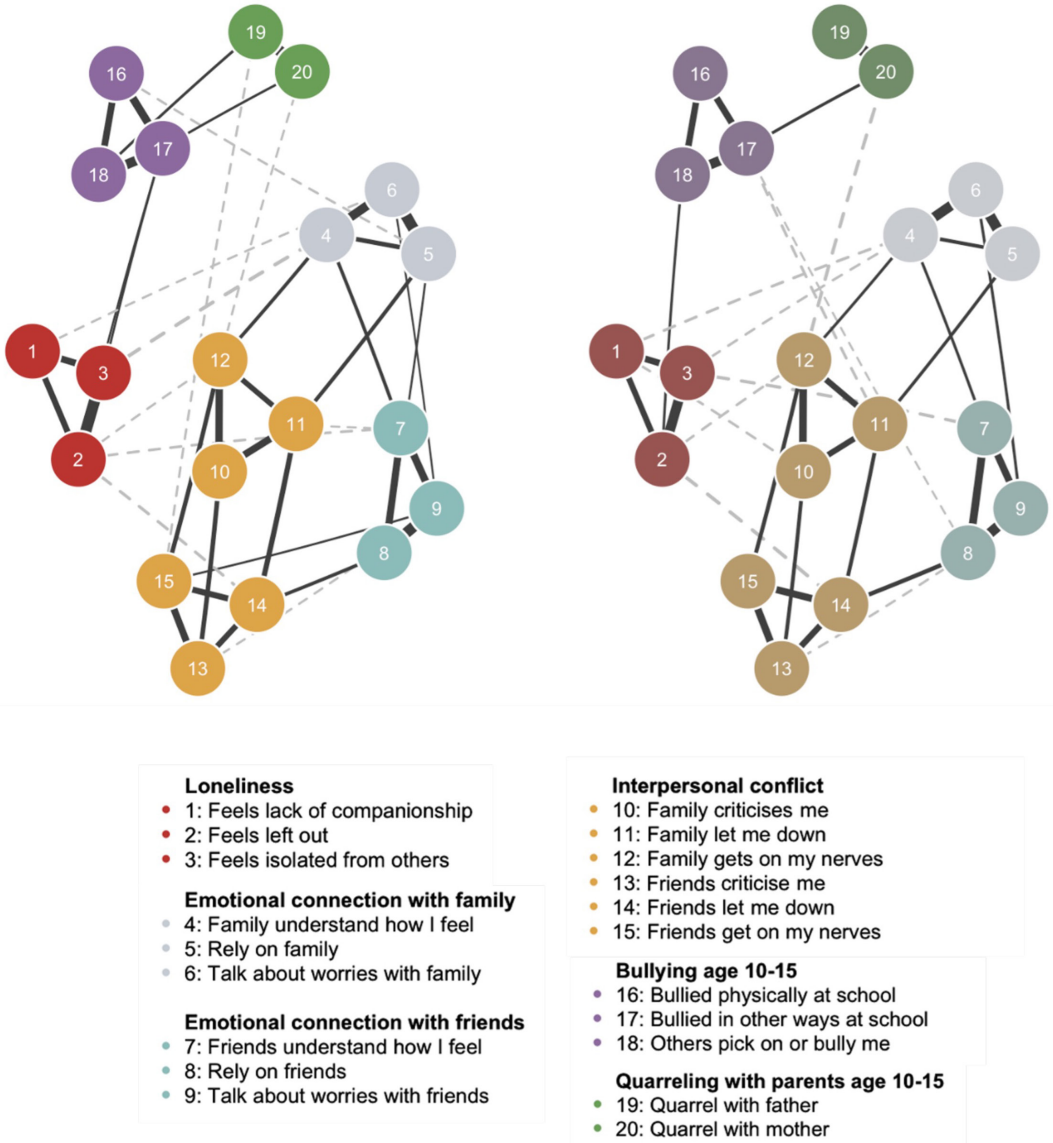
Networks of bullying and family conflict age 10–15 years, loneliness and social connection age 16–24 years for high (left) and low (right) socioeconomic marginalisation groups.

Feeling left out was more strongly associated with feeling isolated in the high group (w=0.52) compared with the low group (w=0.46; *T=*0.094, p=0.023). Feeling less criticised by family was more strongly associated with feeling less let down by family in the high group (w=0.31) compared with the low group (w=0.25; *T*=0.091, p=0.039), and feeling less let down by family was more strongly associated with feeling less let down by friends in the high group (w=0.21) compared with the low group (w=0.14; *T*=0.097, p=0.032). There were no other significant differences between the edges (*ps*>0.05). Findings should be interpreted cautiously because larger sample sizes tend to yield stronger, more stable edges in the network comparison test and the larger sample here is the high group.^56^ Despite differences in specific edges, there were no differences between groups in the overall network structure or strength: the invariance network test (*M=*0.100, p=0.497) and global strength invariance test (*S*=0.323, p=0.346) were not significant. Networks and weights matrices are presented in figure 2 and online supplemental figure S7, respectively.

To contextualise the results, we compared group demographics (table 1). Participants aged 16–20 years were more likely to be in the high socioeconomic marginalisation group, while those aged 20–23 years were more often in the low group (c^2^(8)= 72.416, p<0.001). This likely reflects life transitions between 16 years and 20 years old (eg, transitioning to university/into employment, moving out of childhood home) and a higher likelihood of being in employment in the later years. Participants identifying as Asian and Black were more often in the high group compared to participants identifying as white (c^2^(4)= 76.608, p<0.001). A significantly higher proportion of participants in the high group lived with their parents compared with the low group (c^2^(1)= 5.09, p=0.024). There were no significant differences in the distribution of sex between groups (c^2^(1)= 0.01, p=0.932).

Finally, Welch’s t-tests with Bonferroni correlations showed the high group reported feeling more let down by family (*M=*3.46, *SD*=0.79 vs *M=*3.57, *SD*=0.71; *t*(1574.7)=−3.46, p<0.001), less able to rely on family (*M*=3.42, *SD=*0.84 vs *M=*3.54, *SD=*0.74; *t*(1614)=3.56, p<0.001), and more severe non-physical peer bullying at age 10–15 years (*M*=1.24, *SD*=0.45 vs *M=*1.17, *SD=*0.37; *t*(1708)=4.33, p<0.001). There were no other significant differences, including in the loneliness items (*p_bonf_>0*.05; online supplemental figure S2).

Overall, there were no significant differences in the structure or global strength of the networks between high and low socioeconomic marginalisation groups. Some associations between social connection factors were stronger in the high socioeconomic marginalisation, despite this group reporting feeling more let down and less able to rely on family. This group also reported higher rates of bullying, was younger, more likely to identify as Asian or Black and more often lived with parents than the low socioeconomic marginalisation group.

### Sensitivity checks

As the data wave spans the COVID-19 pandemic (data were collected from January 2019 to May 2021), we conducted a sensitivity analysis to check for the impact of the pandemic on the network of loneliness and social connection. We separated participants into two groups: participating prior to the first lockdown in the UK (participating on or before 29 February 2020; n=1591) and participating after the first lockdown (participating after 29 February 2020; n=940). We then fit the network to each group and compared them using the Network Comparison Test: there were no significant differences in network structure (*M*=0.089, p=0.585) or in global strength (*S*=0.150, p=0.598). The networks are visualised in online supplemental figure S8 in the supplementary materials.

Additionally, we compared across three groups of socioeconomic marginalisation (low, middle and high). The pattern of results was the same as the two groups. After correcting for multiple comparisons, there were no significant differences between low and high (invariance: *M=*0.134, p=0.166; global strength: S=0.534, p=0.028), low and middle (invariance: *M=*0.113, p*=*0.446; global strength: *S=*0.209, p*=*0.349) and middle and high (invariance: *M=*0.094, p*=*0.756; global strength: *S=*0.325, p*=*0.321).

## Discussion

In this study, we used network analysis to explore links between socioeconomic marginalisation, past peer bullying and family conflict (age 10–15 years), social connection with family and friends and loneliness (age 16–24 years) in the UKHLS dataset. Six clusters emerged: loneliness, emotional connection with family, emotional connection with friends, interpersonal conflict, past peer bullying (age 10–15 years) and past family conflict (age 10–15 years). In line with our hypotheses, distinct facets of loneliness related to specific facets of social connection and past bullying. The most central items were talking about worries with family, relying on friends, talking to friends about worries and past non-physical bullying. Some relationships were stronger in the high socioeconomic marginalisation group, which also included more adolescents identifying as Asian or Black, more living with parents and more severe experiences of bullying. Findings demonstrate the ongoing importance of both family and friend connections in late adolescence, the role of past bullying, and highlight potential targets for future confirmatory research and prevention measures.

Our results suggest a trade-off between family and friend connection rather than a simple reorientation towards peers. For example, feeling less understood by family was associated with feeling more able to talk to friends, and feeling criticised by family was associated with feeling more understood by friends. These relationships are not directional; however, this is more nuanced than the social reorientation hypothesis suggests. The social reorientation model conceptualised adolescence as a shift or reorientation from family to friends.^60^ The social reorientation model has been critiqued for limited and unrepresentative evidence.^61^ Some studies have found no change in the amount of time spent with family in adolescence.^62^ Social preference or sensitivity might vary depending on individual differences, situational demands like caring responsibilities and cultural differences.^63^,^65^ Our findings instead support flexible dynamics where adolescents turn to relationships that feel more supportive. This underscores the protective role of both family and friend relationships against loneliness up to the age of 24 years.

We show that socioeconomic context is related to emotional connection with friends and family in adolescence. First, loneliness rates did not differ across socioeconomic marginalisation groups on the UCLA-3 items, contrasting evidence from the previous literature.^18 19 21 23 25^ However, this is in line with a recent review showing no significant correlations between SES factors and loneliness in children and adolescence.^26^ The mixed findings could be due to methodological differences such that previous studies used scales or composite scores of loneliness and included measures of SES that do not capture marginalisation. Item-level analysis revealed differences in social connection. Stronger ties between feeling left out and isolated and between family/friend connections items emerged in the high socioeconomic marginalisation group. Caution is warranted as this group was larger. Still, our hypothesis that feeling left out would be more central in the high socioeconomic marginalisation group was supported. Demographic differences may contextualise these results: participants in the high group were more likely to identify as Asian or Black and more likely to live with parents. These findings align with research identifying close-knit family roles in some cultures. For example, research with Pakistani adolescents reported adolescents spend similar amounts of time with both friends and family outside of school, and activities often involved helping family members.^63^ This suggests that family and friend connections may be protective in different contexts. Following this exploratory, hypothesis-generating analysis, research should test these relationships in a mediation analysis with longitudinal data to confirm this suggestion.

Socioeconomic marginalisation may also increase exposure to bullying, which could be linked to later loneliness and fragmented social connection. In the full sample, non-physical bullying (aged 10–15 years) was linked to feeling more left out and isolated (aged 16–24 years) and the high socioeconomic marginalisation group reported more severe bullying. The results presented here are non-directional; confirmatory research is needed to confirm the direction. The findings are in line with theoretical accounts framing loneliness as a social justice issue that draw attention to the structural factors that shape loneliness and connection.^28^ On a practical level, the research findings presented here show that past bullying, feeling left out, feeling isolated and feeling let down by friends and family might be useful targets for support, especially in those who are experiencing socioeconomic marginalisation. To inform policy, public health intervention studies testing the role of family and friend-based connection strategies and their impact on later loneliness are needed.

There are strengths and limitations to consider in the interpretation of these results. Network analysis is advantageous in that it is data-driven and exploratory, making it useful in generating hypotheses for future confirmatory research. We leveraged a large-scale, population-based UK cohort with data collected at both aged 10–15 years and aged 16–24 years. The breadth of items included in this study has extended previous research seeking to understand the relationship between SES and loneliness using composite scales or single items. Moreover, we developed an intuitive socioeconomic marginalisation score using publicly available data, which could be leveraged in future replications or other studies. On the other hand, associations should be interpreted carefully, as the causal pathways cannot be determined from this analysis. It is also important to interpret the results in the context of the variables measured: previous research has shown that centrality estimates of items can vary depending on the number of items included.^66^ The conclusions here are limited to the variables included in the network and do not extend to social connection and loneliness beyond these variables, for example, the intensity of loneliness or social connection with intimate partners. Moreover, other interpersonal exclusion factors beyond quarrelling with parents and bullying should be included in future research. The findings are also limited to participants who had completed all measures due to listwise deletion in network analysis; this could raise selection bias issues given that items were self-reported. This sample was recruited from the UK and is designed to be representative of the UK population; generalisability outside of the UK context is limited, and the study should be replicated in other UK and non-UK contexts. We used gross household income to make use of publicly available data on gross household income in the UK and to capture socioeconomic marginalisation relative to a participant’s local community. Future studies should investigate these relationships in the context of other SES measures, such as education or perceived wealth.

In summary, our findings highlight a complex interplay between family, friends, bullying and loneliness in adolescence. Talking about worries with family and friends, relying on friends and past non-physical bullying were central. Some connections were stronger in high socioeconomic marginalisation groups, underscoring the impact of the socioeconomic context. Results challenge the social reorientation hypothesis, showing family connections remain important in late adolescence. Past bullying and social connection with family and friends should be tested in future confirmatory research and present promising prevention targets, especially for adolescents experiencing socioeconomic marginalisation.

## Supplementary material

10.1136/bmjph-2025-004053 online supplemental file 1

## Data Availability

Data are available in a public, open access repository.
