# Supplementary material for: Both family and peer relationships matter in late adolescence: a network analysis of loneliness and social connection in a UK cohort
Source: BMJ Public Health. 2026 May 21;4(2):e004053. doi: 10.1136/bmjph-2025-004053 (PMC13202035; doi:10.1136/bmjph-2025-004053)

**Supplementary materials**

***Both family and peer relationships matter in late adolescence: A network analysis of loneliness and social connection in a UK cohort***

**Authors:** Kathryn E. Bates^1^, Lara Acosta Silijeström^1^, Amanah Bokari^1^, Leandra Gyekye^1^, Jennifer Y.F. Lau^1,2^, Lauren Turner^1,2^, Delia Fuhrmann^1^

**Corresponding author:** Dr. Kathryn E. Bates, [kathryn.2.bates@kcl.ac.uk](mailto:kathryn.2.bates@kcl.ac.uk)

**Affiliations:** 1. Department of Psychology, Institute of Psychiatry, Psychology and Neuroscience, King’s College London, UK. 2. Youth Resilience Unit, Queen Mary University.

**Supplementary Methods 1**

The University of Essex Ethics Committee has approved all data collection on Understanding Society main study. COVID-19 surveys and innovation panel waves, including asking consent for all data linkages except to health records. Requesting consent for health record linkage was approved at Wave 1 by the National Research Ethics Service (NRES) Oxfordshire REC A (08/H0604/124), at BHPS Wave 18 by the NRES Royal Free Hospital & Medical School (08/H0720/60) and at Wave 4 by NRES Southampton REC A (11/SC/0274). Approval for asking consent for health record linkage and for the collection of blood and subsequent serology testing in the March 2021 wave of the COVID-19 study was obtained from London – City & East Research Ethics Committee (21/HRA/0644). Approval for the collection of biosocial data by trained nurses in Waves 2 and 3 of the main survey was obtained from the National Research Ethics Service (Understanding Society – UK Household Longitudinal Study: A Biosocial Component, Oxfordshire A REC, Reference: 10/H0604/2). The biosocial data collection at IP12 ‘Understanding Society Health Innovation Panel: Biomeasure and health data collection from the Innovation Panel of the UK Household Longitudinal Study’ was approved by East of England – Essex Research Ethics Committee, Ref 19/EE/0146.

**Table S1**

Glossary of statistical terms

| **Term** | **Explanation** |
| --- | --- |
| Open Science Framework | A free, open platform to support research and collaboration. Researchers store information in projects to provide preregistration of methods and analyses prior to conducting their research, as well as analysis scripts and additional materials. <https://osf.io/> |
| RStudio | A programme for statistical computing and graphics. |
| Package | Extensions of the R statistical language. Packages contain collections of functions, data and code to support data visualisation, data management and data analysis. |
| Bootstrapping | A resampling methods involving repeatedly drawing random samples from an existing dataset and calculating a statistic for each resample. |
| Confidence intervals | A range of values within a specific level of confidence measuring certainty around a sample estimate. |
| Setting the seed | This specifies the random number generate with a specific value to ensure that random number sequences will be reproduced in future computing. |
| Nodes | Individual items/variables entered into a network analysis. |
| Edges | The term used to describe the associations between nodes after controlling for all other associations in the network. |
| Bridges | The nodes that are most connected to nodes from other clusters or dimensions of the network. |

**Figure S1**

Pattern of missing data


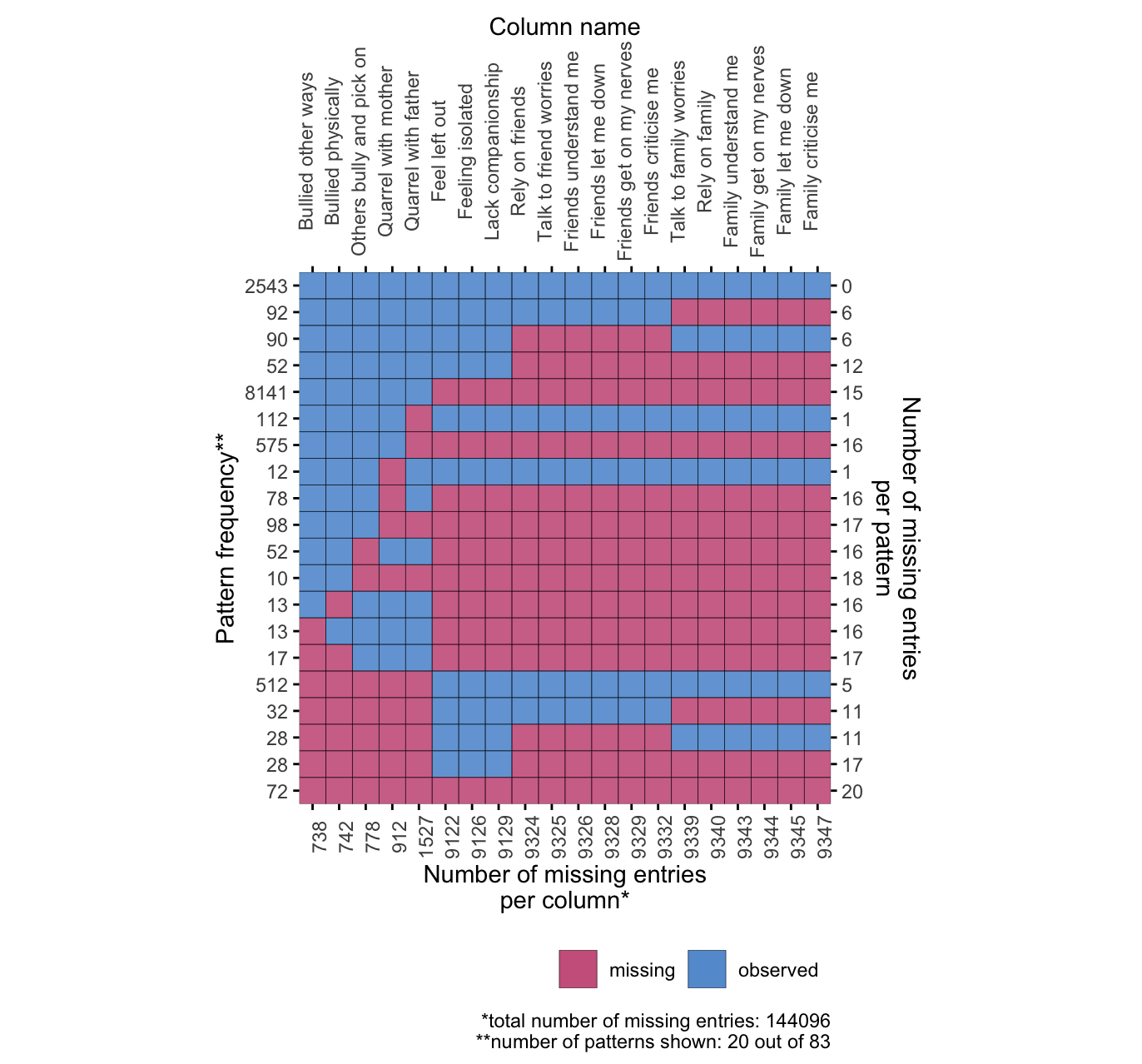


*Note:* This plot indicates missing and observed data and the patterns of missing data. For example, the first row indicates 2543 participants had data for all variables, the fifth row from the bottom indicates that 512 participants had data for all variables apart from prior victimisation (bullied other ways, bullied physically, others bully and pick on, quarrel with father, quarrel with mother). Note that the final sample was 2,531 because 12 participants z scores in physical bullying exceeded +/- 5. Columns are ordered according to number of missing entries per column.

**Figure S2**

Mean and standard error of all items in the network split by high and low economic marginalisation groups. Positively phrased items (understand, rely on, worries) reverse score so that for all variables, high score indicates higher social connection. SEM = socio-economic marginalisation.

**
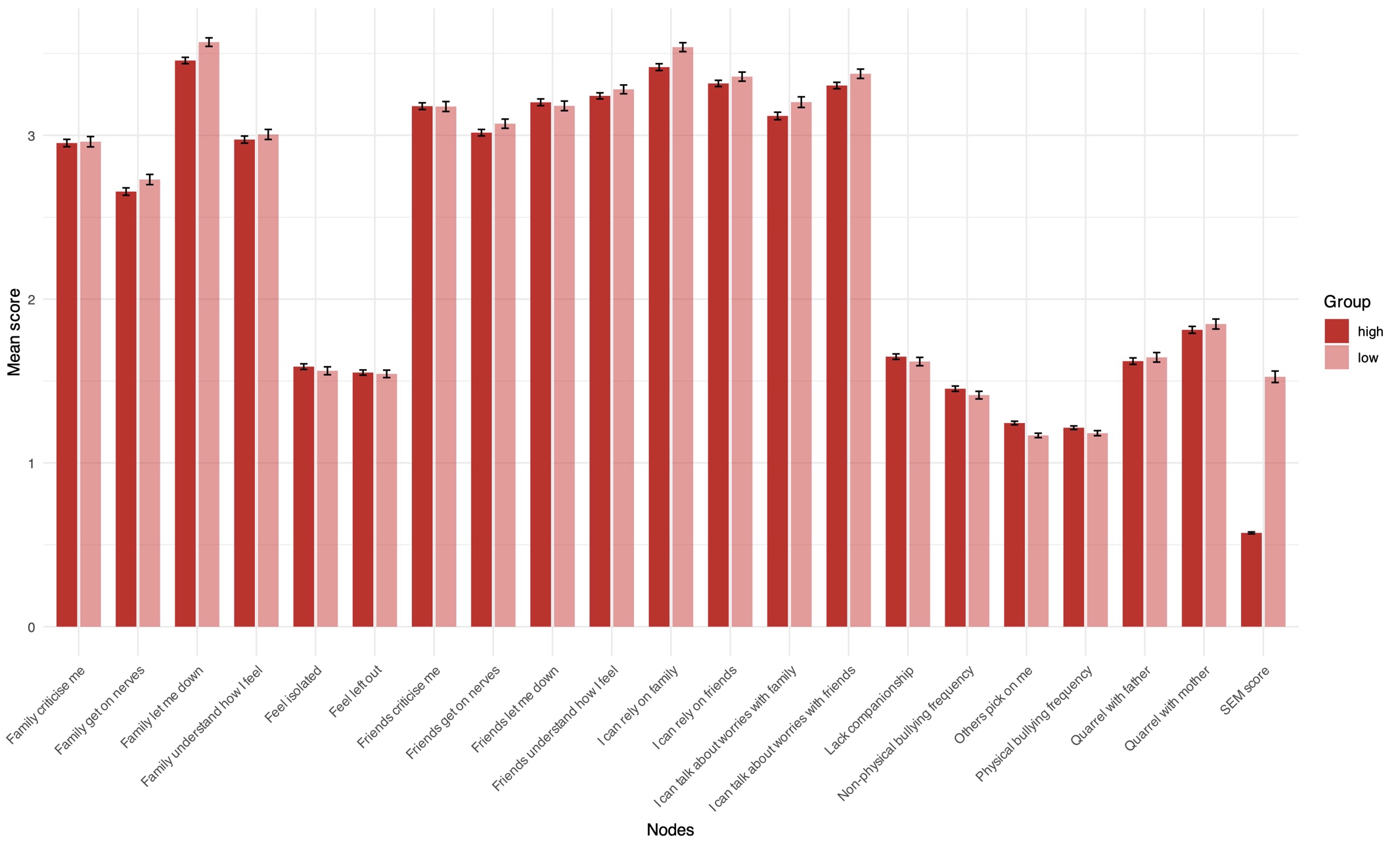
**

**Figure S3**

Map of local authority districts in England, Wales, Scotland and Northern Ireland. Source: Office for National Statistics licensed under the Open Government Licence v.3.0. Contains OS data © Crown copyright and database right 2025.


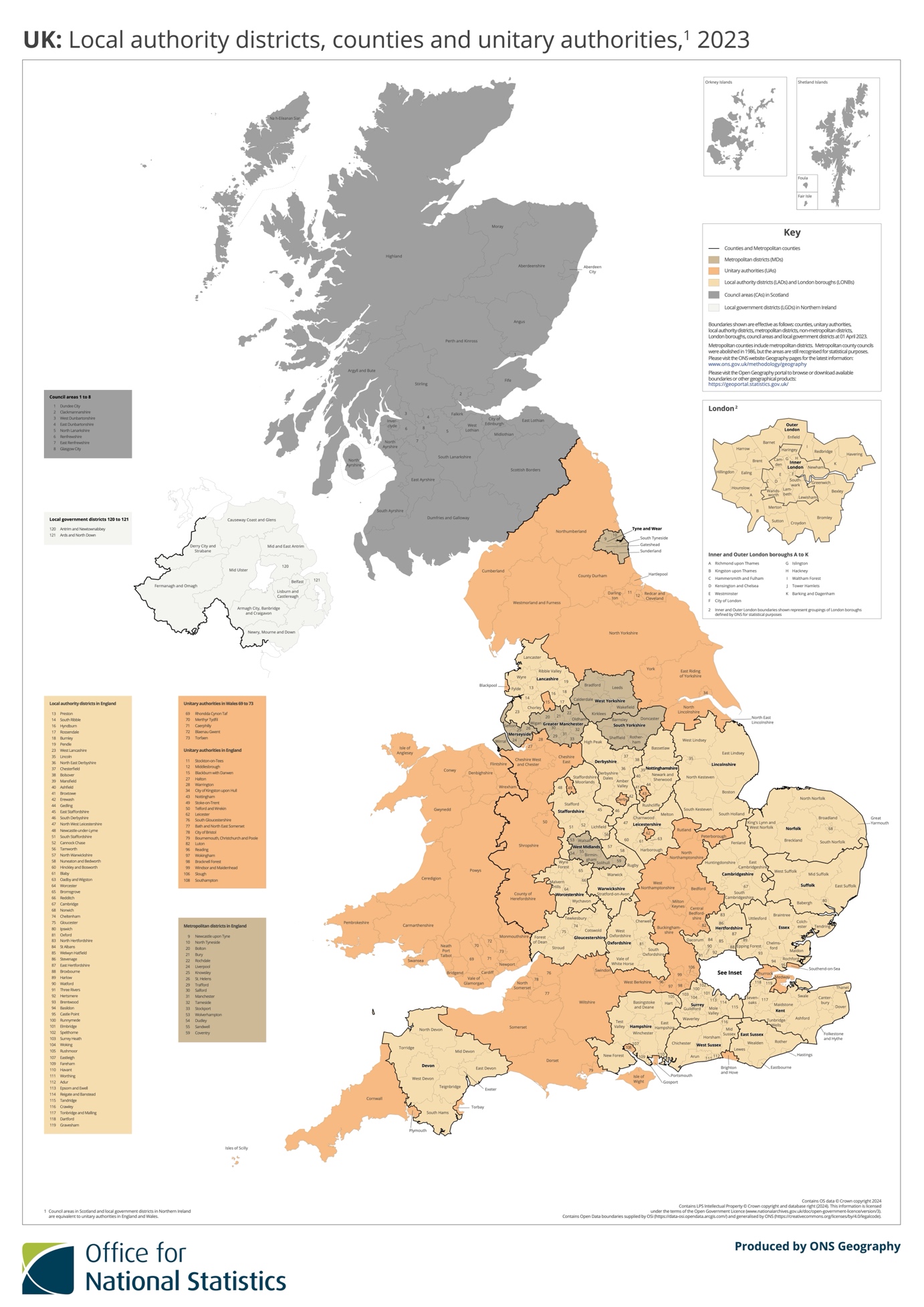


**Figure S4**

Accuracy of edge weights


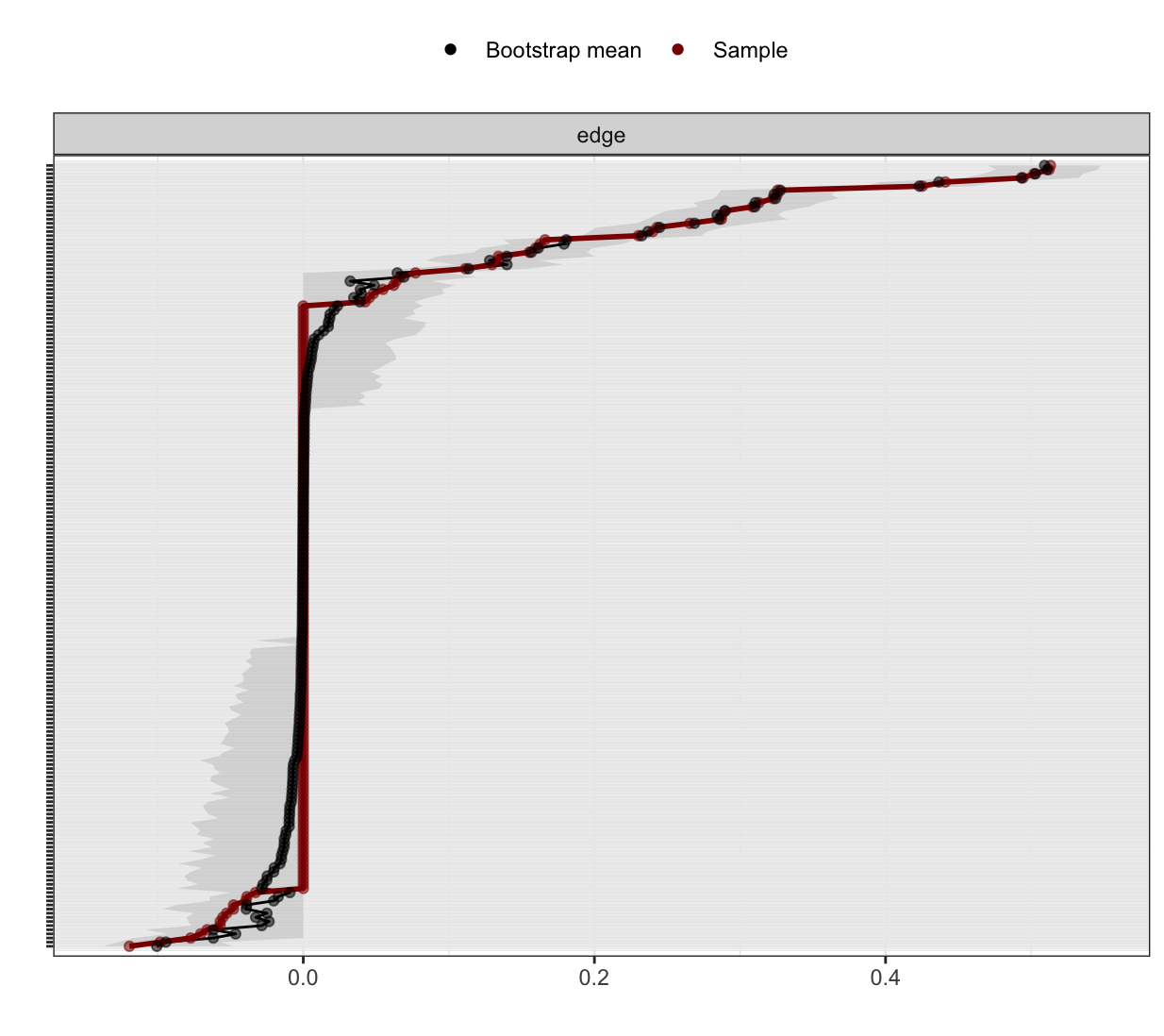


*Note:* The y-axis shows all possible edges from lowest to highest weight. The black line represents average edge weights from the bootstrapped samples and the red line represents edges weights from the sample data. Darker grey lines represent 95% confidence intervals.

**Figure S5**

Centrality statistics from left to right panels: strength, closeness, betweenness, expected influence. Presented in order of expected influence.


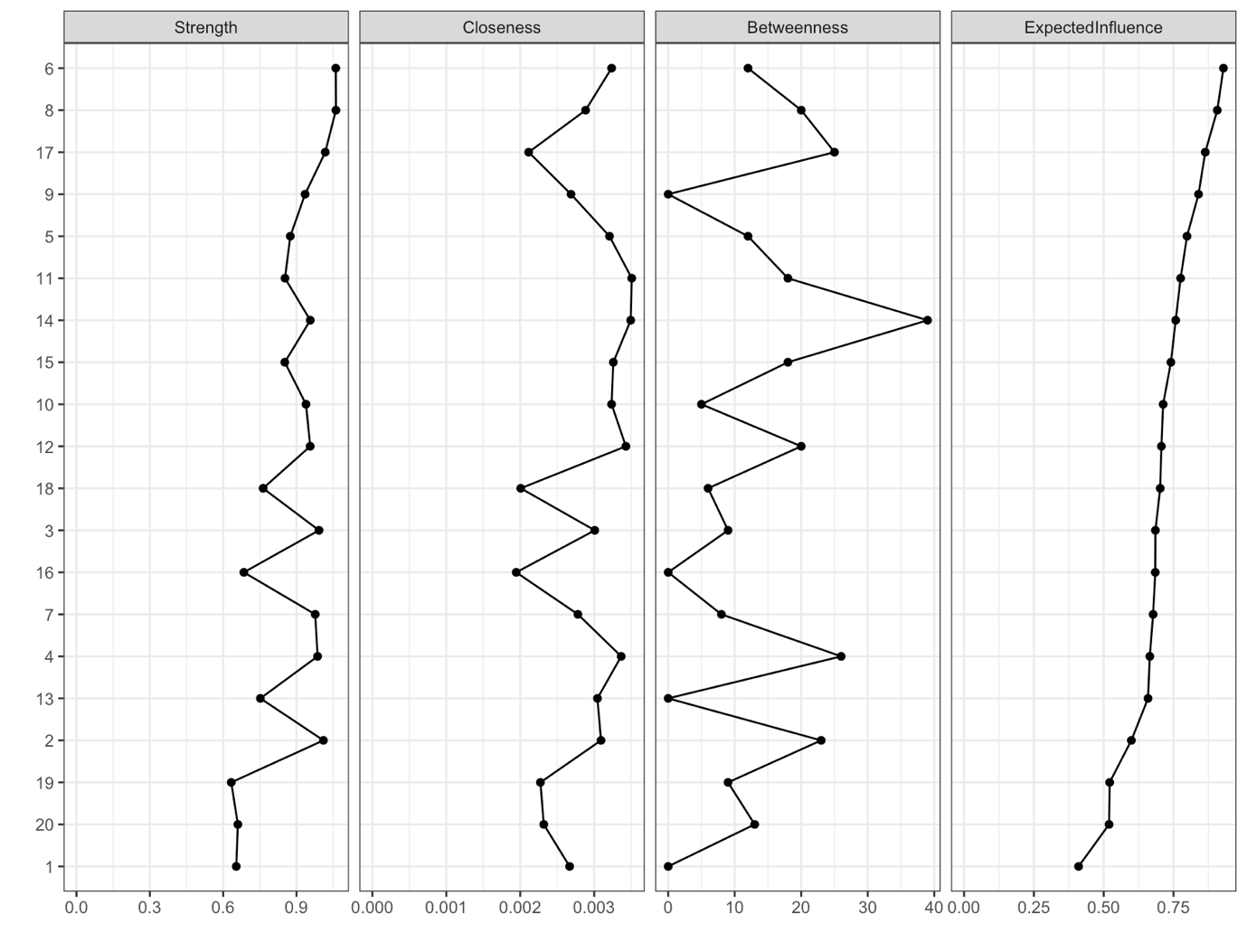


**Figure SX**

Proportion plots for sex, ethnicity, living with parents, age

*Note:* 1 = Lack of companionship, 2 = Feels left out, 3 = Feels isolated from others, 4 = Family understand how I feel, 5 = Rely on family, 6 = Talk about worries with family, 7 = Friends understand how I feel, 8 = Rely on friends, 9 = Talk about worries with friends, 10 = Family criticises me, 11 = Family let me down, 12 = Family gets on my nerves, 13 = Friends criticise me, 14 = Friends let me down, 15 = Friends get on my nerves, 16 = Bullied physically at school age 10-15 years, 17 = Bullied in other ways at school age 10-15 years, 18 = Others pick on or bully me age 10-15 years, 19 = Quarrel with father, 20 = Quarrel with mother.

**Figure S6**

Edge weight’s matrix of full sample network. 0 indicates no edge between two items.


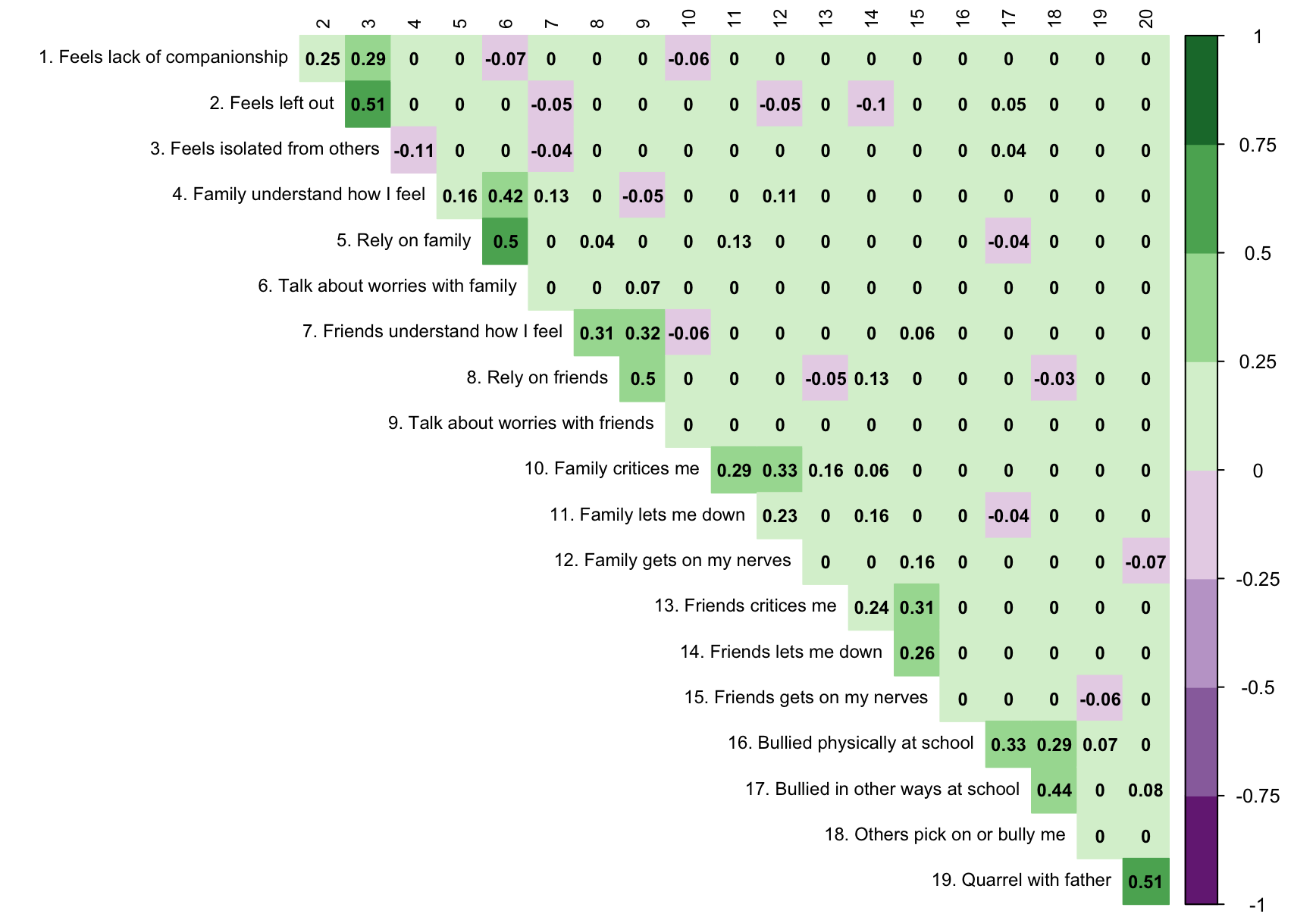


**Figure S7**

Edge weights matrices from networks for high (top) and low (bottom) socio-economic marginalisaton groups


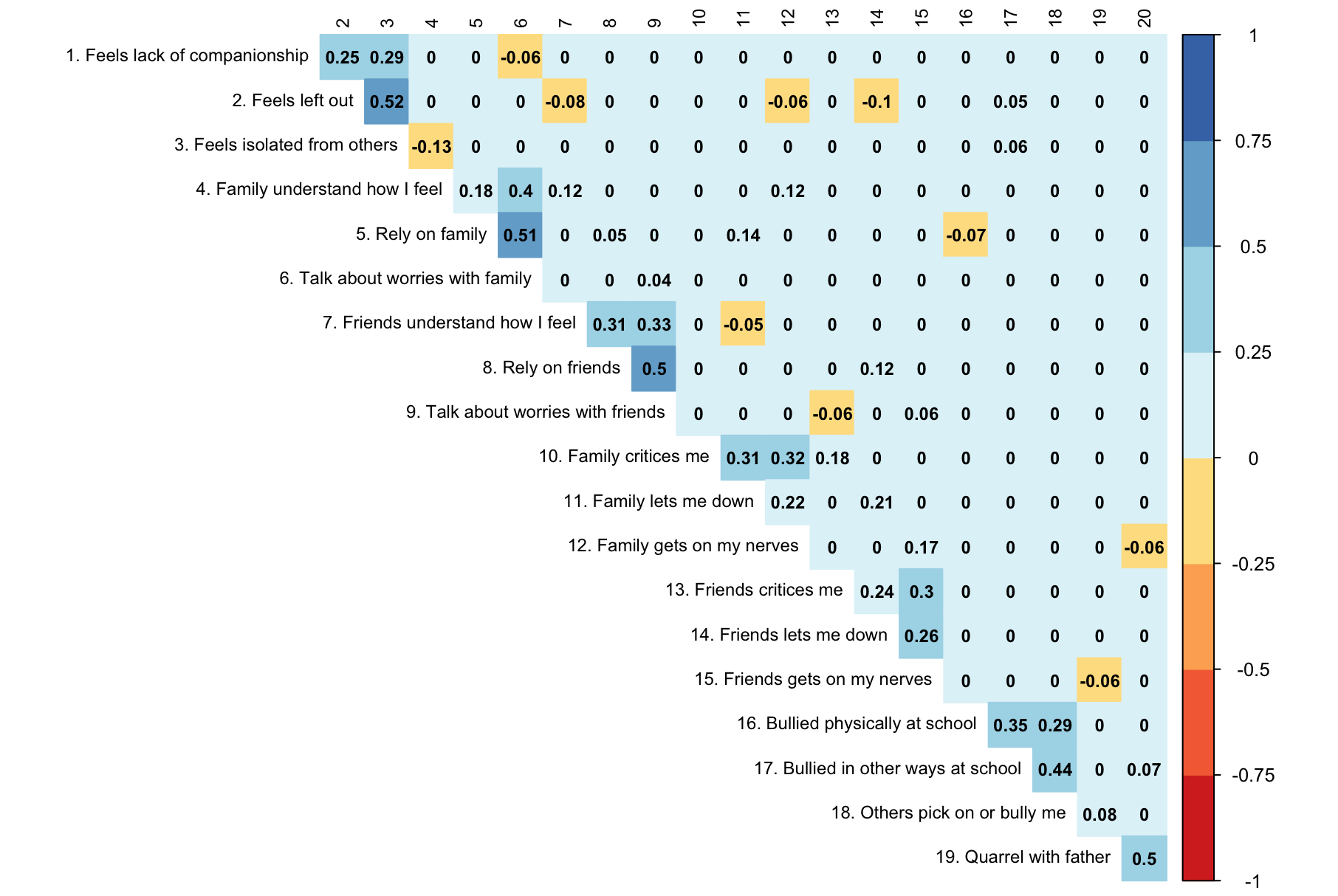


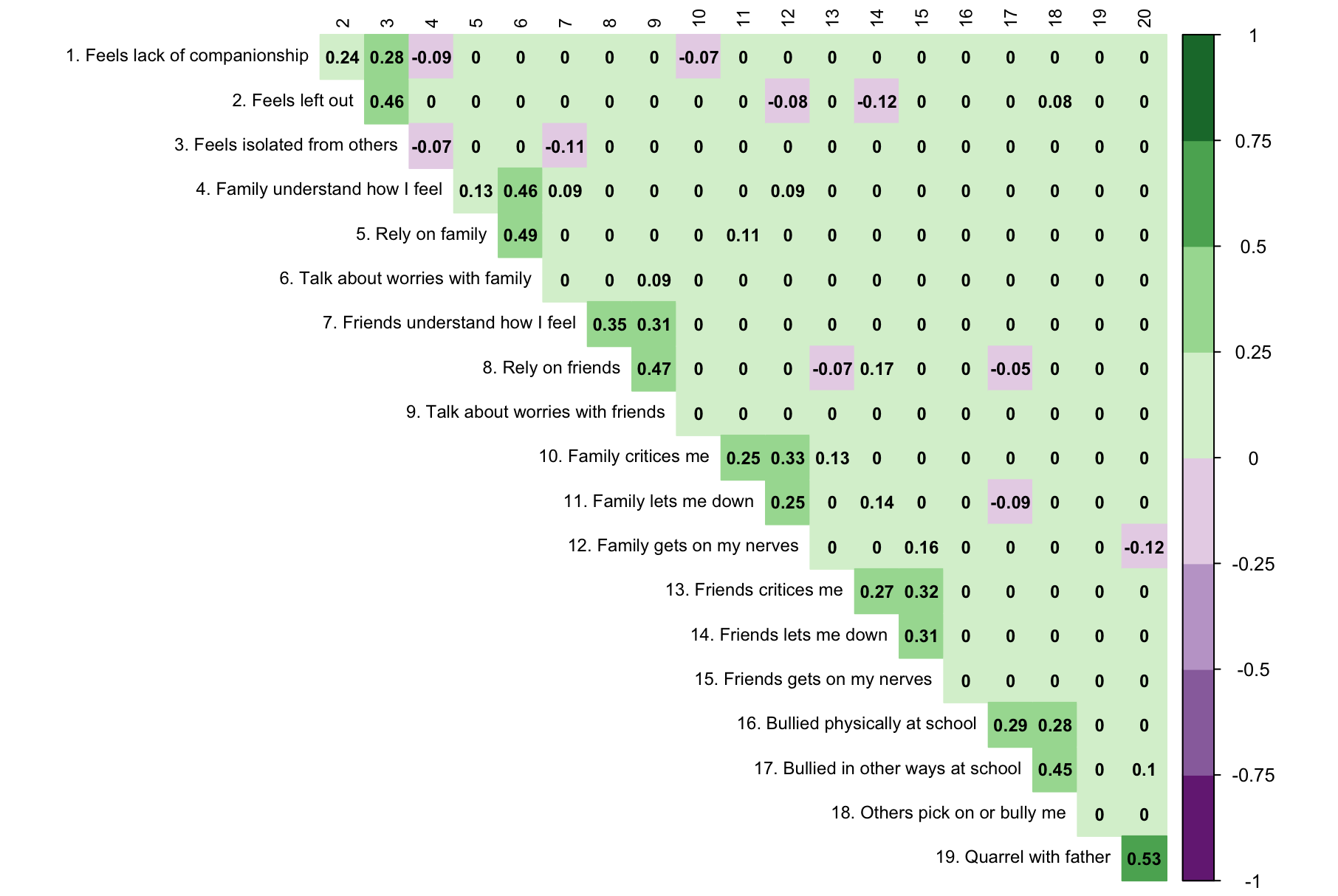


**Figure S8**

Networks for pre-COVID (left) and post-COVID (right) groups.


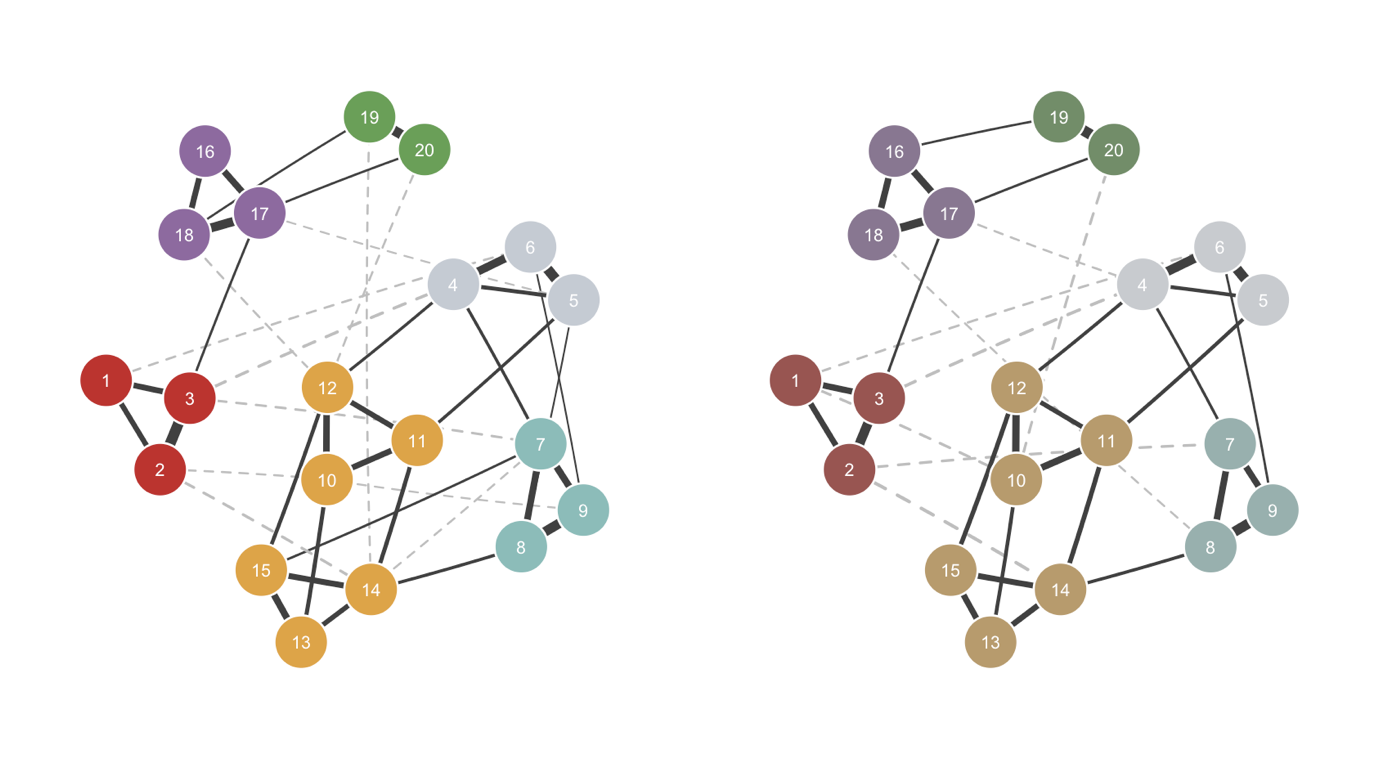


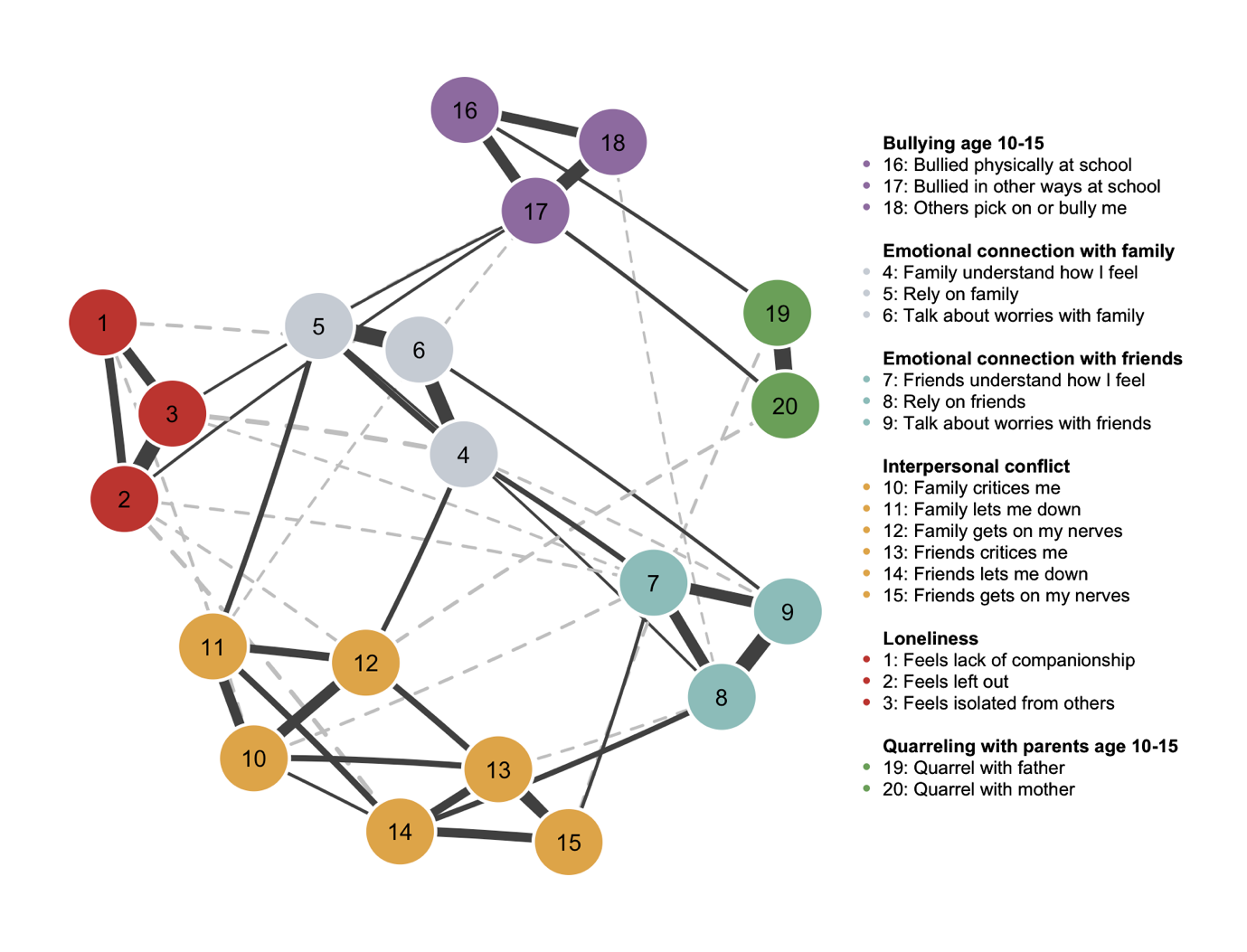

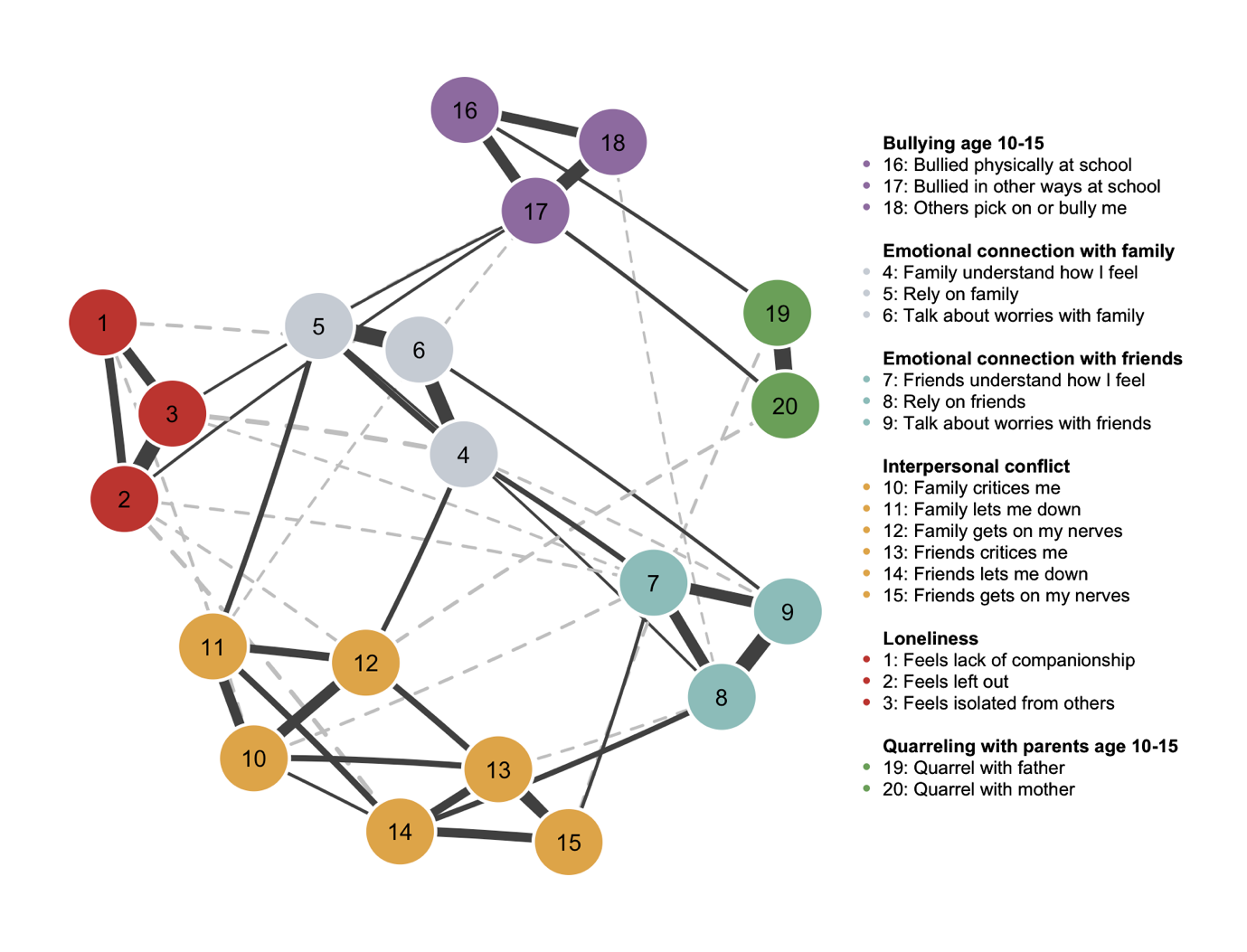

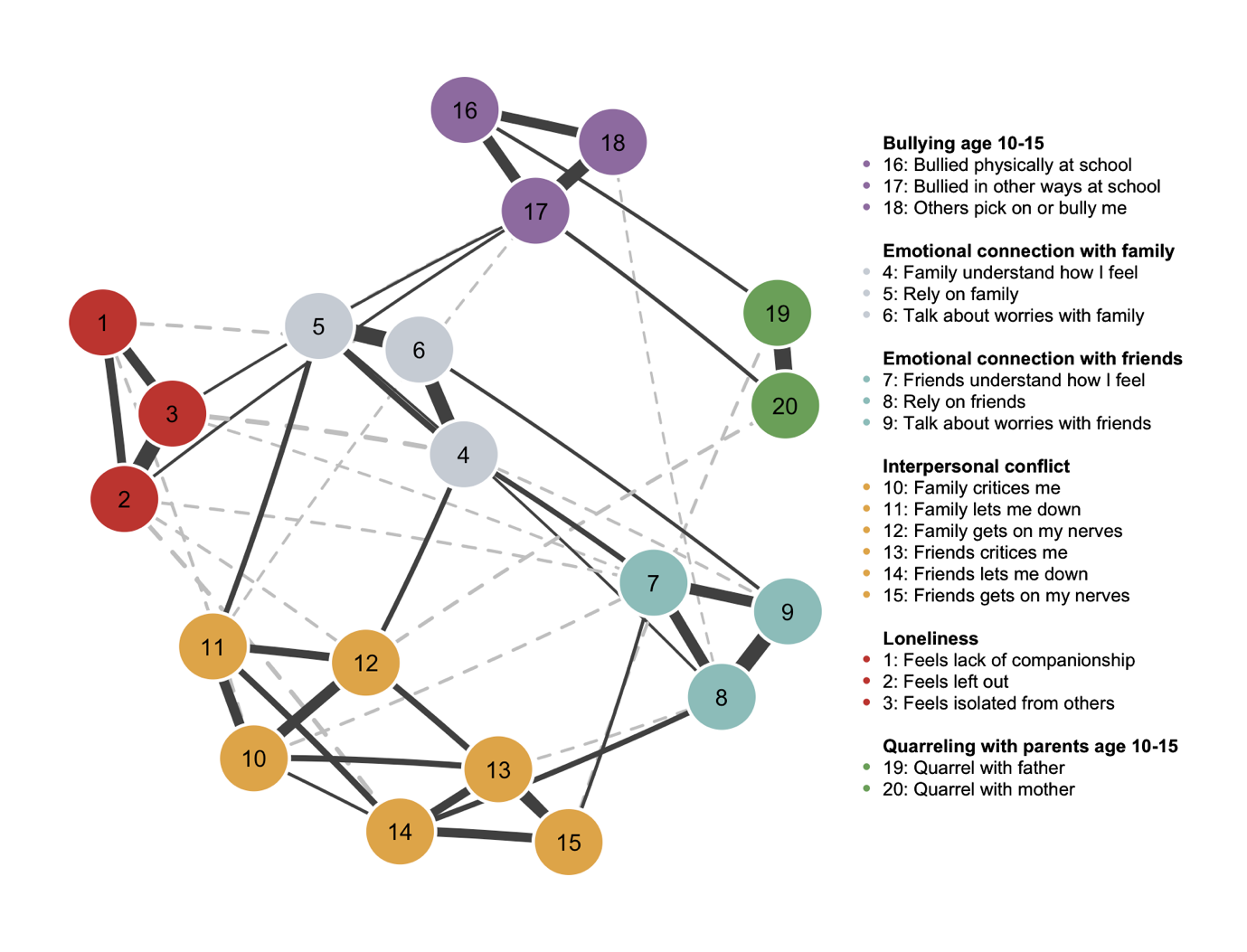

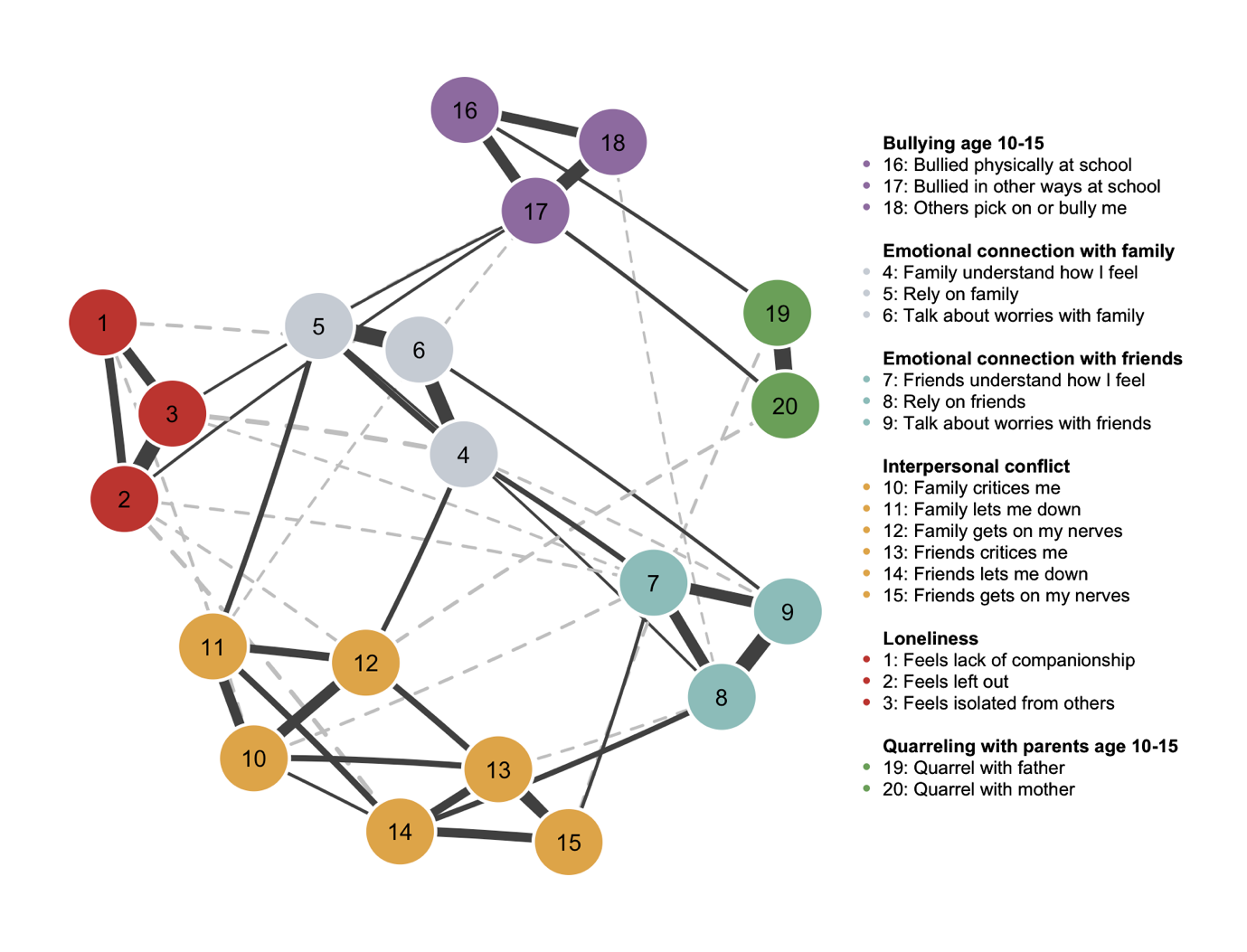

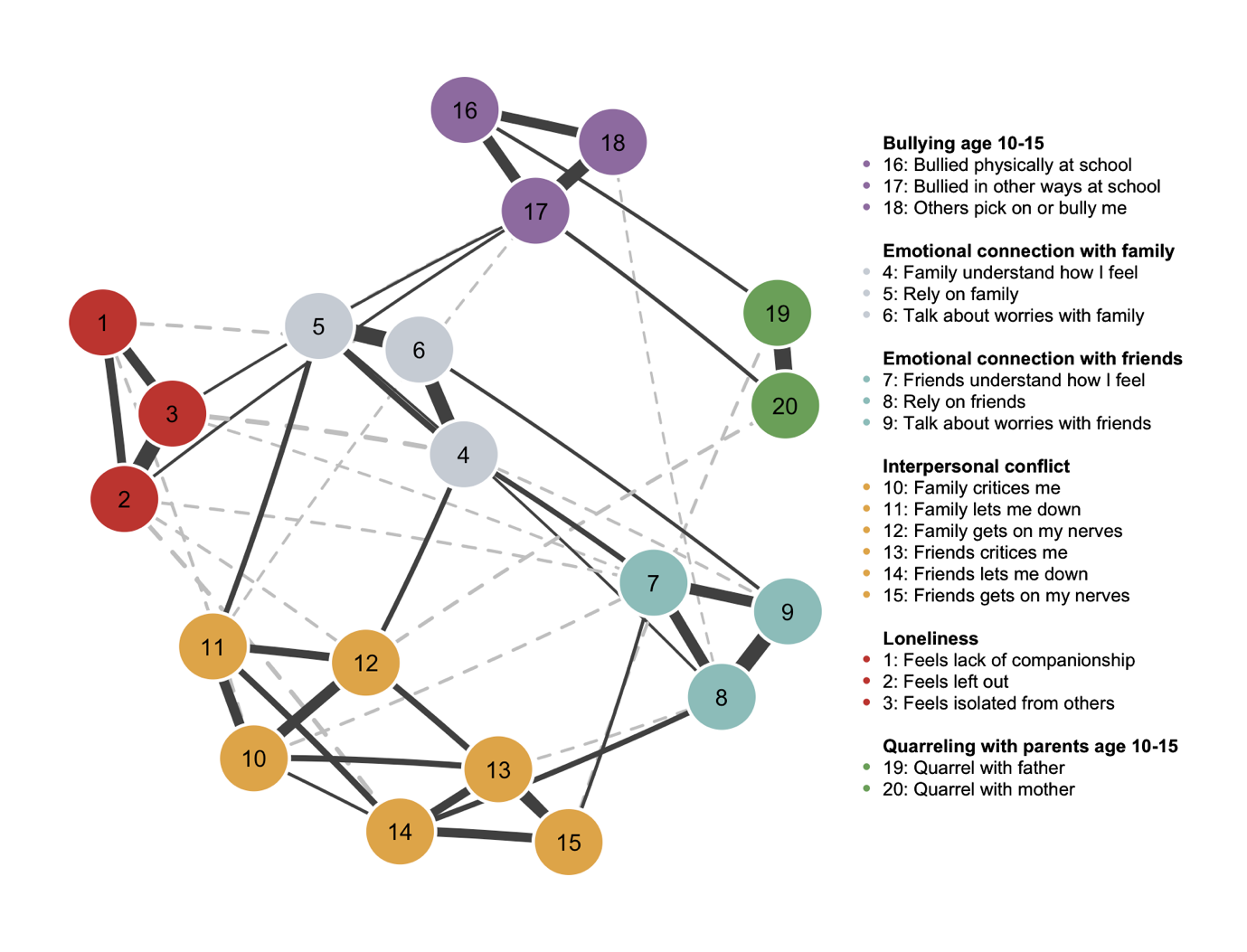

Supplement: online supplemental file 1 [file bmjph-4-2-s001.docx]
